# Effects of generic substitution on refill adherence to statin therapy: a nationwide population-based study

**DOI:** 10.1186/s12913-014-0626-x

**Published:** 2014-12-05

**Authors:** Henrik Trusell, Karolina Andersson Sundell

**Affiliations:** Section of Social Medicine and Epidemiology, Department of Public Health and Community Medicine, Institute of Medicine, The Sahlgrenska Academy at University of Gothenburg, PO Box 453, 405 30 Gothenburg, Sweden; Nordic School of Public Health, Gothenburg, Sweden

**Keywords:** Medication adherence, Generic substitution, Statins, Lipid-lowering therapy

## Abstract

**Background:**

Several countries have introduced generic substitution, but few studies have assessed its effect on refill adherence. This study aimed to analyse whether generic substitution influences refill adherence to statin treatment.

**Methods:**

Between 1 July 2006 and 30 June 2007, new users of simvastatin (n = 108,806) and atorvastatin (n = 7,464) were identified in the Swedish Prescribed Drug Register . The present study included atorvastatin users as an unexposed control group because atorvastatin was patent-protected and thus not substitutable. We assessed refill adherence using continuous measure of medication acquisition (CMA). To control for potential confounders, we used analysis of covariance (ANCOVA). Differences in CMA associated with generic substitution and generic substitution at first-time statin purchase were analysed.

**Results:**

Nine of ten simvastatin users were exposed to generic substitution during the study period, and their adherence rate was higher than that of patients without substitution [84.6% (95% CI 83.5-85.6) versus 59.9% (95% CI 58.4-61.4), p < 0.001]. CMA was higher with increasing age (60–69 years 16.7%, p < 0.0001 and 70–79 years 17.8%, p < 0.0001, compared to 18–39 years) and secondary prevention (12.8%, p < 0.0001). CMA was lower among patients who were exposed to generic substitution upon initial purchase, compared to those who were exposed to a generic substitution subsequently [80.4% (95% CI 79.4-90.9) versus 89.8% (88.7-90.9), p < 0.001]. This difference decreased when those with only one statin purchase were excluded.

**Conclusions:**

Statin refill adherence was higher among patients who exposed to generic substitution compared to those who were not. Increasing age and previous cardiovascular disease affected refill adherence.

## Background

Based on register data on dispensed medicines, previous studies reported an indirect measure of statin refill adherence totalling more than 80% [1,2]. Refill adherence is a valid estimate of adherence to medication [3]. Good adherence to statin treatment associates with significantly lower risks for cardiovascular disease (CVD) and mortality [4].

Although few studies have explored whether generic substitution affects refill adherence, some reports suggest that patients find it more difficult to keep track of their medication after generic substitution [5,6]. However, a small proportion of patients reported intake errors following generic substitution [5], most commonly they simultaneously used two different products with the same active pharmaceutical ingredient. Some reports express concern that generics have lower quality than brand-name products [7,8]. However, a survey reported that only a small group of the patients thought that generics caused more side effects than their brand-name equivalent [8].

Studies investigating whether generic medication and generic substitution affect refill adherence report that adherence is higher among patients who start treatment with generics, compared to those who start with brand-name drugs [7,9]. We wanted determine whether this pattern of adherence applies to refill adherence for statins, using a method validated in the Swedish setting. Thus, this study used continuous measure of medication acquisition (CMA) to analyse whether generic substitution influences refill adherence to statin therapy. We also aimed to assess to what extent socioeconomic and clinical characteristics affect refill adherence.

## Methods

### Study population and study period

Between 1 July 2006 and 30 June 2007, new users of simvastatin (ATC code C10AA01) and atorvastatin (ATC code C10AA05) were identified in the Swedish Prescribed Drug Register (SPDR) [10]. During the study period, index date was defined as the date each individual purchased a statin for the first time. Thus, a new user was defined as an individual who did not fill any prescriptions for lipid-lowering treatment (ATC-code C10) during the 12 months preceding the index date. The present study included atorvastatin as an unexposed control group because no generic substitute for atorvastatin was available during the study period. We followed all participants until death, emigration, or for a maximum of two years after the index date. If an unrecorded death occurred during follow up, we assumed that death occurred on the 15th of the specified month (n = 22), or on July 1st if only the year was specified (n = 2).

We excluded individuals who (i) were younger than 18 years of age at index date; (ii) received multi-dose dispensed drugs during the study period; (iii) were registered as deceased prior to the index date; (iv) purchased more than one statin substance on their index date (n = 15); or (v) died or emigrated on their index date (n = 2). Switching from simvastatin to another statin was allowed because such switches are a normal part of clinical practice. However, we excluded individuals who switched statin substances four or more times during the study period (n = 252).

### Data sources

SPDR data on dispensed drugs included all prescriptions dispensed in Sweden during the study period [10], and information about (i) the dispensed drug (name, substance, drug type, strength, and amount); (ii) the patient (age, sex, and place of residence); (iii) the prescriber (profession and workplace); (iv) the price paid by the patient; (v) the level of reimbursement; and (vi) the date of issue and dispensing. Recorded as free text, dosage instructions sometimes include the indication. When the pharmacy dispensed a product other than the one prescribed (due to generic substitution), information about both products is provided.

Information about participants’ highest attained level of education and migration background were collected from the Swedish Longitudinal Integration Database for Health Insurance and Labour Market Studies (LISA) on individual-level. We obtained information about hospital care (i.e., diagnosis codes), which was classified according to the International Statistical Classification of Diseases and Related Health Problems 10th revision (ICD-10) from the Swedish National Patient Register.

### Exposure assessment

We defined generic substitution as not receiving the prescribed product, but receiving a substitute product approved by the Medical Products Agency. Generic substitution can also occur if the prescribed product is unavailable at the pharmacy. In Sweden, the prescriber and the pharmacy can restrict generic substitution due to medical reasons or issues related to formulation, dosage regimen, etc. In that case, the pharmacy dispenses the prescribed product and the Pharmaceutical Benefit Scheme (PBS) covers the full cost included in the patients total co-payment [11]. If the patient refuses substitution, the pharmacy attributes the price of the cheapest available substitutable product to the total PBS co-payment and the patient pays the balance out of pocket. We considered same-strength products from the same manufacturer identical even if the package size differed. However, we considered different strengths from the same manufacturer as different products because pharmacists cannot dispense other strength than that prescribed.

We classified exposure to generic substitution into four groups: (i) simvastatin users who experienced generic substitution during follow-up, (ii) simvastatin users who did not experience generic substitution due to an active decline by the prescriber/pharmacist or the patient, (iii) simvastatin users who did not experience generic substitution but where no active decline was made (i.e., no cheaper substitute was available), and (iv) atorvastatin users.

### Outcome measure

We calculated refill adherence using CMA [i.e., (number of days’ supply dispensed during the study period)/(number of days in the study period)].

A previous study reported that 98.4% of new simvastatin users took one tablet or capsule per day [2]. Therefore, our assumed dosage was one tablet or capsule per day. We accounted for stockpiling of medication before the previous supply was fully consumed by adding the remaining amount to the next purchase. When statin substances were switched, we disregarded the remaining amount of the previously purchased substance.

To study whether generic substitution occurring sometime during the study period affected CMA, we divided the study population into the four groups described above.

To study whether timing of initial generic substitution affected refill adherence, we divided simvastatin users who had experienced generic substitution into two subgroups: (i) first generic substitution at index date, and (ii) first generic substitution later on.

Two sensitivity analyses assessed the potential impact of early discontinuation and switching on CMA. First, we analysed the potential impact of early discontinuation by excluding participants who filled only one prescription. Second, we investigated the impact of switching by analysing participants who did not switch and those who switched (with one to three statins) separately.

### Potential confounders

Potential confounders included age, sex, level of education, migration background, number of prescription drugs used, pharmacologically treated diabetes, and statin prescribed for secondary prevention (i.e., diagnosed renal disease and/or CVD). After determining age at index date, we divided participants into five age groups: 18–39, 40–49, 50–59, 60–69, and 70–79 years of age.

We grouped highest attained level of education into primary school, secondary school, and university degree. We defined migrant background according to Statistics Sweden’s alternative classification, wherein an individual has migrant background if he/she was born outside Sweden to two parents also born abroad, or born in Sweden to two parents born abroad. If we could not determine the birthplace of one or both parents, we assumed that the parent(s) was born in the same place as the studied individual.

We defined the number of prescription drugs purchased in the year before the index date as the number of purchased substances. Pharmacologically treated diabetes was defined as having purchased blood glucose-lowering drugs (ATC-code A10) in the year before the index date. We differentiated between insulin only (A10A) and oral antidiabetics (A10B) taken alone or with insulin. We also determined how many generically substituted drugs each individual received.

Diagnoses of CVDs and renal diseases registered five or fewer years before the index date served as indicators for secondary prevention. CVDs included ischaemic heart diseases (ICD-10 code I20-I25), pulmonary heart disease, and diseases of pulmonary circulation (I26-I28), cerebrovascular diseases (I60-I69), and diseases of arteries, arterioles, and capillaries (I70-I79). Renal diseases included glomerular diseases (N00-N08) and codes indicating renal failure (N17-N19) [12].

### Statistical analyses

We compared mean values using the t-test for two groups, and used analysis of variance (ANOVA) with Bonferroni correction to compare unadjusted means between more than two groups. To compare adjusted means at the 95% confidence level, accounting for potential confounders, we used analysis of co-variance (ANCOVA) with Bonferroni correction. We calculated 95% confidence intervals (95% CI) in the ANOVA and ANCOVA analyses. P-values <0.05 were considered statistically significant. All statistical analysis was conducted using SAS statistical software version 9.3 (SAS Institute, Cary, NC, USA).

### Ethical considerations

The study was approved by the Regional Ethics Committee in Gothenburg (registration reference: 284–09).

## Results

### Study population

Among 116,270 participants, 108,806 were new users of simvastatin and 7,464 were new users of atorvastatin (controls). Altogether, 241 simvastatin users emigrated and 3,090 died during follow up; among these, 120 emigrations and 1,579 deaths occurred during the first year of follow up. Among atorvastatin users, 45 emigrated and 170 died during follow up whereof 24 emigrated and 85 died within the first year after index date. About half of simvastatin and atorvastatin users were women (47.0% versus 45.4%), and a majority had Swedish background (83.2% versus 78.3%) (Table 1). The proportion with a previous CVD diagnosis among simvastatin and atorvastatin users was 23.2% and 17.1%, respectively.

**Table 1:** **Socioeconomic and clinical characteristics of new users of simvastatin (n = 108,806) and atorvastatin (n = 7,464)**

	**Simvastatin**		**Atorvastatin**	
**Variable**	**n**	**%**	**n**	**%**
**Gender**				
Female	51,130	47.0	3,389	45.4
Male	57,676	53.0	4,075	54.6
**Age (years)**				
18–39	2,482	2.3	284	3.8
40–49	9,696	8.9	779	10.4
50–59	25,398	23.3	1,963	26.3
60–69	37,303	34.3	2,549	34.2
70–79	25,224	23.2	1,531	20.5
80–	8,703	8.00	358	4.8
**Background**				
Swedish	90,517	83.2	5,844	78.3
Migrant	18,289	16.8	1,620	21.7
**Place of birth if foreign background**				
Sweden	756	4.1	85	5.2
Other Nordic country	6,495	35.5	452	27.9
Other EU27 country	3,567	19.5	373	23.0
Other European country	2,886	15.8	229	14.1
Africa	482	2.6	43	2.7
North America	186	1.0	28	1.7
South America	473	2.6	55	3.4
Asia and Oceania	2,872	15.7	296	18.3
Former Soviet Union	115	0.6	14	0.9
Unknown place of birth	457	2.5	45	2.8
**Level of education**				
Primary school, <9 years	19,199	17.7	1100	14.7
Primary school, 9 years	9,180	8.4	707	9.5
Secondary school	40,097	36.9	2862	38.3
University, <2 years	2,962	2.7	234	3.1
University, ≥2 years	16,621	15.3	1405	18.8
Postgraduate	714	0.7	87	1.2
Unknown education level	20,033	18.4	1069	14.3
**Previous disease**				
Cardiovascular disease	25,266	23.2	1,278	17.1
Renal disease	388	0.4	93	1.2
Both	492	0.5	40	0.5
**Pharmacological diabetes treatment**				
Oral antidiabetics	9,798	9.0	581	7.8
Insulin	4,553	4.2	400	5.4
Combination of these	3,056	2.8	229	3.1
Total	17,407	16.0	1,210	16.2

The mean number of prescription drugs purchased one year before an individual’s index date was 4.8 (SD 4.8) for simvastatin users ( median = 4.0). For atorvastatin users, the mean was 5.3 (SD 5.5) and the median 4.0. During follow up, the mean number of generically substituted statins was 2.6 (SD 2.2) for simvastatin users and 1.9 (SD 2.2) for atorvastatin users. The median was 2.0 and 1.0, respectively.

### Refill adherence

For simvastatin, the mean CMA was 84.3% (SD 107.0), with a median of 95.1%. For atorvastatin, the mean CMA was 79.0% (SD 55.9), with a median of 85.0%.

Among simvastatin users, 92.8% were exposed to generic substitution during follow up, and 1.6% purchased brand-name simvastatin products during follow up. The adjusted mean value of CMA was significantly lower for all individuals without generic substitution (i.e., groups ii, iii, and iv together), compared to individuals who were exposed to substitution [59.9% (95% CI 58.4-61.4) versus 84.6% (95% CI 83.5-85.6), p <0.001]. The adjusted mean CMA was lowest among simvastatin users not exposed to generic substitution [36.7 (95% CI 34.8-38.7)] (Table 2). Furthermore refill adherence was significantly lower among simvastatin users who actively refused generic substitution, compared to simvastatin users exposed to generic substitution and atorvastatin users.

**Table 2:** **Mean values of CMA (95% confidence intervals) from ANOVA and ANCOVA analyses**

	**Unadjusted values**			**Adjusted values**		
	**N**	**Mean (%)**	**95% CI**	**n**	**Mean (%)**	**95% CI**
Simvastatin users with generic substitution	100,995	86.6	86.1 – 87.1	82,532	84.6	83.5 – 85.6
Simvastatin users with active of generic substitution	801	72.2	68.9 – 75.4	651	71.6	66.5 – 76.7
Simvastatin users not exposed to generic substitution	7,010	52.8	45.8 – 59.8	5,590	36.7	34.8 – 38.7
Atorvastatin users	7,464	79.0	77.7 – 80.3	6,395	79.0	77.1 – 80.9
*For those with generic substitution*						
Generic substitution at the first purchase	64,314	83.5	82.7-84.2		80.4	79.4-90.9
Generic substitution later on	36,681	92.2	91.8-92.5		89.8	88.7-90.9

In the total study population, CMA was higher among older individuals, individuals with previously diagnosed CVD and type 2 diabetes medicines (Table 3). CMA was significantly lower in individuals with migrant background, compared to individuals with Swedish background.

**Table 3:** **Analysis of CMA by covariates in relation to generic substitution (n = 95,168)**

**Parameter**	**Difference in estimate (%)**	**p-value**
**Gender**		
Female	(reference)	–
Male	1.6	0.0004
**Age (years)**		
18–39	(reference)	–
40–49	8.3	<.0001
50–59	13.0	<.0001
60–69	16.7	<.0001
70-79	17.8	<.0001
80–	-	-^a^
**Level of education**		
Primary school	(reference)	–
Secondary school	−0.3	0.6
University	−0.3	0.6
**Background**		
Swedish	(reference)	–
Migrant	−4.4	<.0001
**Drugs prior to index**		
0	(reference)	–
1	−0.8	0.3
2–4	−0.1	0.9
5–	1.8	0.003
**Previous cardiovascular disease**		
No	(reference)	–
Yes	12.8	<.0001
**Pharmacological diabetes treatment**		
None	(reference)	–
Insulin only	0.0	1.0
Oral antidiabetics only, or both insulin and oral antidiabetics	3.6	<.0001

Differences in continuous measure of medication acquisition (CMA) by exposure to generic substitution by each covariate analysed with ANCOVA. The first class of each covariate is used as a reference ( N = 95,168, whereof 88,773 new simvastatin users).

^a^Data on educational level was not available for individuals 80 years and older. Thus, this age group was excluded from the analysis.

Altogether, 10,673 simvastatin users and 848 atorvastatin users filled their prescription only once. CMA was somewhat higher after excluding such participants, but the results did not change substantially (Table 4). CMA did not increase among simvastatin users who were not exposed to generic substitution. Comparing those who switched between statins to those who did not, CMA was higher for simvastatin users who actively refused generic substitution and simvastatin users who did not experience generic substitution; it was lower among atorvastatin users (Table 4).

**Table 4:** **Sensitivity analyses of CMA (95% confidence intervals) by number of filled statin prescriptions and switching between statins**

	**Number of filled prescriptions**			**Switching between different statins**					
	***≥ 2 filled statin prescriptions***			***No switches***			***1-3 switches***		
	**N**	**Mean (%)**	**95% CI**	**N**	**Mean (%)**	**95% CI**	**N**	**Mean (%)**	**95% CI**
*Entire study population*									
Simvastatin users with generic substitution	94525	90.1	89.9-90.3	93572	86.9	86.0-87.8	7423	83.1	82.0-84.1
Simvastatin users with active decline of generic substitution	697	79.8	84.7-86.8	533	68.1	56.4-79.9	268	80.2	74.6-85.7
Simvastatin users not exposed to generic substitution	2911	52.5	76.6-83.0	5651	49.3	45.7-52.9	1292	75.9	73.4-78.5
Atorvastatin users	6616	85.8	50.9-54.0	6172	79.6	76.2-83.1	1359	67.0	64.6-69.5
*Those with generic substitution*									
Generic substitution at first purchase	57844	88.7	88.4-89.1	59175	83.7	83.0-84.4	5139	80.7	79.6-81.8
Generic substitution later on	36681	92.2	91.8-92.5	34297	92.4	91.4-93.4	2284	88.4	86.8-90.1

### Adherence in relation to timing of generic substitution

CMA was lower among simvastatin users who were exposed to generic substitution at first purchase, compared to users who were exposed subsequently (Table 2). Among those who were exposed to generic substitution, CMA was significantly higher among older individuals, those previously diagnosed with CVD, individuals with oral antidiabetic medicines, and individuals taking at least five medications (Table 5). CMA was significantly lower among individuals with migrant background, compared to individuals with Swedish background.

**Table 5:** **Analysis of CMA by covariates in relation to timing of generic substitution among those who experienced generic substitution (n = 82,532)**

**Parameter**	**Difference in estimate (%)**	**p-value**
**Gender**		
Female	(reference)	–
Male	0.7	0.07
**Age (years)**		
18–39	(reference)	–
40–49	8.6	<.0001
50–59	13.3	<.0001
60–69	16.9	<.0001
70–79	17.6	<.0001
80–	-	-^a^
**Level of education**		
Primary school	(reference)	–
Secondary school	−0.1	0.8
University	−0.02	1.0
**Background**		
Swedish	(reference)	–
Migrant	−4.1	<.0001
**Drugs prior to index**		
0	(reference)	–
1	0.1	0.9
2–4	0.6	0.3
5–	2.8	<.0001
**Previous cardiovascular disease**		
No	(reference)	–
Yes	11.2	<.0001
**Pharmacological diabetes treatment**		
No	(reference)	–
Insulin only	−1.2	0.2
Oral antidiabetics only or both insulin and oral antidiabetics	3.7	<.0001

Differences in continuous measure of medication acquisition (CMA) for those who experienced generic substitution at first purchase, compared to subsequent substitution by each covariate, analysed with ANCOVA. The first class of each covariate is used as a reference (N = 82,532).

^a^Data on educational level was not available for individuals 80 years and older. Thus, this age group was excluded from the analysis.

Excluding those with only one statin purchase yielded a smaller difference in CMA between those who experienced generic substitution at first purchase and those who did so later on (Table 4).

## Discussion

More than nine of ten new simvastatin users experienced generic substitution during the follow up period. Refill adherence was higher among individuals experiencing substitution, comparing to those who did not. Simvastatin users who actively declined substitution showed higher refill adherence, compared to participants who accepted substitution. Increasing age and statin use for secondary prevention associated with higher refill adherence.

Adherence levels for simvastatin concurred with results from Denmark [1], but were lower than those reported in a previous Swedish study [2], partly due to different exclusion and inclusion criteria. The effects of generic substitution on adherence are consistent with previous quantitative [7,9,13] and qualitative [6] studies reporting that generic substitution did not negatively affect adherence level. A reason may be that individuals who experience substitution may receive more attention at the pharmacy [9]. However, few studies have assessed the effects of generic substitution and other policy measures, and the potential for considerable differences between therapeutic areas requires further research. Refill adherence does not measure actual intake of a drug, making it relevant to combine adherence with other measurements to further validate our findings. We chose CMA to assess refill adherence because it depicts possession of a pharmaceutical and has proven reliable in a similar setting [2].

We defined generic substitution as receiving a product other than that specified on the prescription. However, we were unable to identify either the number of actual substitutions or the number products dispensed during follow up. Studies analysing differences in adherence to generics and brand-name products concluded that receiving generics did not in itself affect adherence level [7,14].

Because switching statins is part of clinical practice, we did not exclude such patients, and we allowed them to fill prescriptions for other statin substances subsequently, during follow up. We assessed whether switching affected refill adherence during sensitivity analyses. Early discontinuation or non-initiation was neither a reason for exclusion since excluding those who filled only one prescription would overestimate refill adherence [15]. Factors other than generic substitution (e.g., side effects) can explain early discontinuation and non-initiation [16] and result in lower refill adherence. To assess such impact, we included those factors in the sensitivity analyses, which showed no evidence that non-initiation or early discontinuation affected the differences in refill adherence estimates for those who did not experience generic substitution. As expected, refill adherence increased for the group exposed to generic substitution at the index date. Also, by allowing switching between statins, part of the discontinuation is handled. Prescriptions filled close to date of death or emigration increase adherence. Previous studies suggested that simultaneous intake of multiple products with the same active ingredient could increase after generic substitution [5] which would hence increase refill adherence. This would also apply to individuals using combination treatment (i.e., prescribing two different-strength tablets). However, such scenarios are unclear in the present data.

We selected new statin users to ensure a uniform study population, where previously developed adherence patterns to statin treatment would not influence the adherence estimate. Thus, we were able to isolate the effects of generic substitution to greater extent. We imposed a 12-month washout period because Swedish prescriptions are valid for one year. Our study group assigned new atorvastatin users to an unexposed control group because atorvastatin was not substitutable during the study period. Both substances were included in the PBS during the study period although atorvastatin had limited reimbursement since Swedish therapeutic guidelines recommended simvastatin as first-line therapy. Atorvastatin was recommended when simvastatin failed to achieve treatment goals or was inappropriate for other reasons. Although treatment indications are similar, given the therapeutic guidelines, atorvastatin users may have more severe problems. In Sweden there is no approval process for prescribing medicines with limited reimbursement provided within the PBS. However, our comparison of background characteristics and medical history could not confirm this, there was similarity of socioeconomic characteristics between simvastatin and atorvastatin users and the presence of pharmacologically treated diabetes. The proportion of pre-existing CVD was higher among simvastatin users, suggesting that statin use for secondary prevention of CVD was more common for simvastatin than atorvastatin. However, selection bias might influence whether an individual received a simvastatin or atorvastatin prescription. Although atorvastatin lacks generic equivalents, parallel imported drugs were available, and these products are often identical and not regarded as substitution.

We excluded individuals younger than 18 years and those who received multi-dose dispensed drugs because they are not always in charge of their own medication. Further, multi-dose drugs are automatically dispensed at regular intervals, creating an artificial adherence pattern and were therefore excluded. Due to the construction of the Swedish PBS [11], stockpiling can occur, contributing to higher CMA. We imposed a two-year follow-up period to reduce the effects of stockpiling.

We were unable to examine primary non-adherence (i.e., not initiating treatment) because SPDR records only dispensed prescriptions, not issued prescriptions. In Sweden primary non-adherence varies from 1.5% for cardiovascular drugs in general to around 7%–8% for statins among stroke patients, based on electronic prescriptions [17,18].

The National Patient Register covers secondary health care, but limits coverage for diagnoses commonly handled in primary care, such as diabetes. Therefore, we used purchased anti-diabetic drugs as a proxy variable for pharmacologically treated diabetes. By separating individuals using dispensed insulin or oral anti-diabetics and those using both, we attempted to distinguish between individuals with type 1 and type 2 diabetes. However, the insulin treatment group included individuals with both types of diabetes. Many patients with diabetes type 2 are treated non-pharmacologically [19], this is thus not a suitable proxy for diabetes itself.

To identify individuals who received statins as secondary prevention, we used most recent guidelines diagnosis codes from the National Patient Register. Importantly, contact with a healthcare provider does not always generate a diagnosis. Therefore, a more comprehensive indicator of statin use for secondary prevention could involve reviewing patient’s medical records and combining diagnoses with data about health procedures (e.g., stents). Because the Patient Register did not include full coverage of procedures during the study period, we were unable to make a determination in this regard. Generic simvastatin is relatively inexpensive. Thus, employment status and income level are less likely to affect results and were not included in our analysis.

## Conclusions

Measured using CMA, mean values of refill adherence to simvastatin and atorvastatin were 84.3% and 79.0%, respectively. Most simvastatin users experienced generic substitution sometime during the study period, and refill adherence was highest among those who had experienced generic substitution. Certain patient characteristics affected adherence level. In particular, refill adherence increased with age and among individuals who had experienced a cardiovascular event. Patients’ concerns about generic substitution may require more information from the prescriber and at the pharmacy.
